# Serum Level of Interleukin 17 in Patients with Erosive and Non erosive Oral Lichen Planus

**DOI:** 10.5681/joddd.2013.016

**Published:** 2013-05-30

**Authors:** Firoz Pouralibaba, Zohre Babaloo, Farzaneh Pakdel, Marziye Aghazadeh

**Affiliations:** ^1^Dntal and Periodontal Research Center, Tabriz University of Medical Sciences, Tabriz, Iran; ^2^Assistant Professor, Department of Oral Medicine, Faculty of Dentistry, Tabriz University, Tabriz, Iran; ^3^Assistant Professor, Department of Immunology, Faculty of Dentistry, Tabriz University, Tabriz, Iran; ^4^Assistant Professor, Department of Oral Medicine, Faculty of Dentistry, Tabriz University, Tabriz, Iran; ^5^Assistant Professor, Department of Oral Medicine, Faculty of Dentistry, Tabriz University, Tabriz, Iran

**Keywords:** Biopsy, interleukin-17, oral lichen planus

## Abstract

**Background and aims:**

Oral lichen planus is a relatively common chronic oral mucosal disease of unknown etiology. Regarding numerous studies on the immunologic factors involved in the etiology of lichen planus, the present study evaluated the serum interleukin-17 (IL-17) level in patients with erosive and non-erosive oral lichen planus.

**Materials and methods:**

This descriptive analytical study included 24 patients with erosive oral lichen planus (EOLP), 24 patients with non-erosive oral lichen planus (NEOLP) and 24 healthy volunteered as control. Blood samples of the subjects underwent ELISA, using special kits, to determine serum interleukin-17 levels. Data was analyzed using with descriptive statistic, chi-square test, and one-way ANOVA and Tukey post-hoc test with SPSS 16 software.

**Results:**

EOLP patients showed a high level of serum IL-17 compared with NEOLP patents and control groups (EOLP=184.16 ± 12.41 pg/mL, NEOLP=106.09±10.78 pg/mL, control=15.50 ± 4.34 pg/mL, P - 0.001).

**Conclusion:**

High level of serum IL-17 in erosive oral lichen planus patients compared to the non-erosive type and healthy individuals may be the reason for higher inflammation and atrophy in the erosive type.

## Introduction

Lichen planus is a relatively common mucocutaneous disease. Lichen planus affects most frequently the oral mucosa^1^. In 15% of the cases, the lesions are only seen in the oral cavity and no cutaneous involvement is present.^2^ Oral lichen planus (OLP) affects one to two percent of the general adult population and is the most common noninfectious oral mucosa disease. The prevalence of oral lichen planus is reported to be 0.5-2%.^3^

Oral lichen planus is seen in two non-erosive (reticular, papular, plaque-like) and erosive froms.^4^ The reticular form is the most common form of lichen planus and usually causes no symptoms., Erosive lichen planus is not as common as the reticular form; however, the lesions are painful, thus important for patients. In some cases, the dorsum of tongue is ulcerated resulting in severe pain and trouble in eating.^4^

Although the etiology and the pathogenesis of the disease is not entirely known, various factors including genetic predisposition, stress, some of viral and bacterial agents may act as risk factors for lichen planus.^3-6^ The role of immune system as a primary factor in the pathogenesis of lichen planus has become clearer in recent years. In the histopathological view, basal layer degeneration and band-like infiltration of T lymphocytes and macrophages are seen. This particular view can be interpreted as a cell-mediated role of the immune system in the pathogenesis of lichen planus.^7^

T helper 1 and T helper 2 are independent subdivisions of T cells. Recognized recently is a third “T helper” subdivision, which can play a principal role in defense against extra-cellular pathogens.^8^ This subdivision of T cells controls immune and inflammatory responses through secretion of cytokaines like Interleukin 17. This family of T cells provides a new route for cooperation between innate and acquired immunity.^9^ The major role of IL-17 is to increase the expression of threatening factors for colony chemokaines, methaloproteinase, and IL-6. Therefore, IL-17 is a strong stimulator for recalling, activating, and immigration of neutrophiles, production of INF-alpha, IL-B from macrophages, and recalling eosinophiles.^9,10^ Considerable evidence is suggestive of the important role of IL-17 family in inflammatory, autoimmune, and cancer diseases. High levels of IL-17 has been found in various human inflammatory diseases such as arthritis rheumatoid, air way infections, asthma, Helicobacter pylori infection, multiple sclerosis, infectious bile disease (IBD), and psoriasis.^11,12^

Evidence is suggestive of the notion that lichen planus is an immunological mucodermal. The aim of the present study was to evaluate Serum interleukin-17 level in patients with erosive and non-erosive oral lichen planus.

## Materials and Methods

A total of 48 patients with OLP (24 erosive and 24 non-erosive sub-type) and 24 age- and sex-matched healthy volunteers were included, which was approved by the ethics committee of Tabriz University of Medical Sciences.

The patients were selected, simple random accessible sampling method, from those referring to the Department of Oral Medicine, Tabriz University of Medical Sciences, Tabriz, Iran, with a clinical or clinical-pathological diagnosis of oral lichen planus without consideration of dysplasia. The study procedure was explained to the patients and a written informed consent was taken from those who agreed to include in the study. Biopsies were taken when necessary. Exclusion criteria included lichenoid reactions e.g. to drugs, acquired or innate immune system deficiencies, contraindication for biopsy (where necessary), presence of active HCV, HIV, or TB infections, and pregnancy. Control subjects were selected with a simple random method from healthy individuals referring to the university clinic for dental treatments.

Venal blood samples (5 mL) were taken from subjects in all groups. Serum IL-17 level was determined using a special kit (Human IL-17, ELISA Crokit ko13207p, South Korea) with ELISA assay.

Data was analyzed with descriptive statistic (mean ± SD, frequency), chi-square test, one-way ANOVA and Tukey post-hoc test using SPSS 16.0 software. P < 0.05 was considered statistically significant.

## Results

Mean age and sex distribution of the subjects in the three study groups was shown in table 1. There were no significant differences in regards to mean age and gender distribution between the study groups (P > 0.05), and therefore, age and gender variables were correctly matched between groups.

**Table 1:** Age and sex distribution of patients evaluated

	EOLP	NEOLP	Control	P –value
Age (years)	33.26 ± 7.52	36.97 ± 6.15	37.06 ± 6.93	0.19
Sex	24 (14male/10female)	24 (12 male /12 female)	24 (13 male /11 female)	0.88

Note: Data are presented as Mean ± SD and frequency; EOLP, Erosive lichen planus; NEOLP, Non-erosive lichen planus.

Mean and standard deviation (SD) of serum IL-17 levels in the three groups are shown in table 2. There was a significant difference between serum IL-17 levels between the groups. Tukey post-hoc test revealed statistically significant differences between all studied groups (P < 0.001).

**Table 2:** Levels of IL-17 in the serum samples from patients (pg/ml)

	EOLP	NEOLP	Control	P –value
Serum IL-17	184.16±12.41	106.09±10.78	15.50±4.34	0.00
	(163male,212female)	(81M,128F)	(9M,25F)	

Data represent the Mean ± SD (Max, Man). EOLP: Erosive lichen planus; NEOLP: Non-erosive lichen planus

## Discussion

In the present study, serum IL-17 level was evaluated in erosive and non-erosive oral lichen planus patients without consideration of dysplasia, as there is no study on the relationship between IL-17 level and dysplasia. The results indicated that serum IL-17 level was higher in patients with both erosive and non-erosive lichen planus compared to healthy individuals. In addition, serum IL-17 level was significantly higher in patients with erosive oral lichen planus compared to those with the non-erosive type.

Possible role of interleukin-17 in the pathogenesis of lichen planus is studied by Shaker & Hassan.^12^ They found that the serum levels of IL-17 were significantly higher in patients compared with controls and IL-17 can contribute to the pathogenesis of lichen planus by enhancing T cell-mediated reactions and inducing production of chemokines and other cytokines. The mean level of IL-17 in lichen planus patients in this study was lower and this could be because of history of taking corticosteroids in the studies patients.

Xie et al^13^ studied implications of Th1 and Th17 cells in pathogenesis of oral lichen planus and suggested Th1 cells in OLP lesions play a crucial role in the activation of cytotoxic CD8+ T cells. The data revealed that IL-17 mediated immune response may correlate with OLP subtypes and play an important role in the pathogenesis of OLP, as the level of this interleukin was lower in the reticular from compared to the erosive form. Although the obtained figures are not consistent with those of the present study, the results are overall supportive of each other. The differences in IL-17 levels in the three studies could be a reflection of different disease stages or genetic differences between Iranian and Chinese populations in producing IL-17 cytokines, as this is dependent on HLA-DR. On the other hand, a different commercial ELISA kit was employed in the present study (Koma Biotech, Human IL-17 core kit) in comparison with that used by Xie et al^13^ (NeoBioscience, China). Different sensitivities of two kits may be another reason for obtained inconsistent figures.

Except these two studies, there was no other study on IL-17 levels and lichen planus; however, IL-17 levels have been studied vastly in different conditions. Hofstetter et al^14^ state that Th-17 cells can act as T effectors cell population in the autoimmune central nervous system disease—Multiple Sclerosis (MS). The observed higher levels of IL-17 in oral lichen planus cases of the present study may indicate the probable role of IL-17 in autoimmune diseases.

In another study evaluating IL-11/IL17 levels in gingival crevicular fluid (GCF) in patients with chronic gingival disease,^15^ the authors demonstrated that IL-17 level increased with higher pocket depth and bone destruction. Vernal et al^16^ also found IL-17 in GCF in patients with chronic periodontitis. These findings also indicate an immunologic role for IL-17 in chronic gingival disease.

In one study, cellular apoptosis level was compared in reticular and erosive oral lichen planus, and the results showed a significantly increased apoptosis in the erosive type and a marked reduction in the thickness of oral epithelium in erosive oral lichen planus compared to the reticular type.^17^ Areas with atrophy were also seen more frequently in the histopathological views of erosive oral lichen planus. Based on the results of the latter study indicating a higher inflammation and cell destruction in erosive OLP, it could be interpreted that as IL-17 increases in inflammatory diseases, it results in secretion of high amounts of pre-inflammatory cytokines, and subsequently it is effective in formation of OLP and even its different presentations.

## Conclusion

IL-17 serum level was higher in patients with erosive and non-erosive oral lichen planus in comparison with healthy patients. IL-17 serum level was significantly higher in erosive oral lichen planus compared to the non-erosive type and healthy individuals. This high level may be the reason for higher inflammation and atrophy in the erosive type.

## References

[1] Farh D, Dupin N (2010). Pathophysiology, etiologic factors, and clinical management of oral lichen planus, part I: facts and controversies. Clinics in Dermatology.

[2] Black MM. Lichen planus and lichenoid disorders. In: Burns T, Breathnach S, Neil C, Griffiths CE (eds). Rook's Textbook of Dermatology, 8th ed. St. Louise: Blackwell Science; 2010: p. 1670-98.

[3] Sugerman PB, Savage NW, Walsh LJ, Zhao ZZ, Zhou XJ, Khan A (2002). The pathogenesis of oral lichen planus. Crit Rev Oral Biol Med.

[4] DeRossi SS, Ciarrocca KN (2005). Lichen planus, lichenoid drug reactions, and lichenoid mucosits. Dent Clin N Am.

[5] Moravvej H, Hoseini H, Barikbin B, Malekzadeh R, MeshkatRazavi G (2007). Association of Helicobacter pylori with lichen planus. Indian J Dermatology.

[6] Lodi G, Carrozzo M, Harris K, Piattelli A, Teo CG, Gandolfo S (2000). Hepatitis C virus-associated oral lichen planus: no influence from hepatitis G virus co-infection. J Oral Pathol Med.

[7] Regezi JA, Scuibba J, Jordan CR ( 2008). Oral Pathology Clinical Pathologic Correlations.

[8] Zhang WP, Chen Y, Geng N, Tian K, Bao DM, Yang MZ (2006). The role of matrix metalloproteinases and their tissue inhibitors in oral lichen planus. Zhonghua Kou Qiang Yi XueZaZhi.

[9] Rhodus NL, Cheng B, Ondrey F (2007). Th1/Th2 cytokine ratio in tissue transudates from patients with oral lichen planus. Mediators Inflamm.

[10] Fouser LA, Wright JF, Dunussi-Joannopoulos K, Collins M (2008). Th17 cytokines and their emerging roles in inflammation and autoimmunity. Immunol Rev.

[11] Afzali B, Lombardi G, Lechler RI, Lord GM (2007). The role of T helper 17 (Th17) and regulatory T cells (Treg) in human organ transplantation and autoimmune disease. Clin Exp Immunol.

[12] Shaker O, Hassan AS (2012). Possible role of interleukin-17 in the pathogenesis of lichen planus. Br J Dermatol.

[13] Xie S, Ding L, Xiong Z, Zhu S (2012). Implications of Th1 and Th17 cells in pathogenesis of oral lichen planus. J Huazhong Univ Sci Technolog Med Sci.

[14] Hofstetter H, Gold R, Hartung HP (2009). Th17 Cells in MS and Experimental Autoimmune Encephalomyelitis. Int MS J.

[15] Yetkin Ay Z, Sutcu R, Uskun E, Bozkurt FY, Berker E (2009). The impact of the IL-11:IL-17 ratio on the chronic periodontitis pathogenesis: a preliminary report. Oral Dis.

[16] Vernal R, Dutzan N, Chaparro A, Puente J, Antonieta Valenzuela M, Gamonal J (2005). Levels of interleukin-17 in gingival crevicular fluid and in supernatants of cellular cultures of gingival tissue from patients with chronic periodontitis. J Clin Periodontol.

[17] Brant JM, Vasconcelos AC, Rodrigues LV (2008). Role of apoptosis in erosive and reticular oral lichen planus exhibiting variable epithelial thickness. Braz Dent J.

